# Thermodynamic analysis and kinetic optimization of high-energy batteries based on multi-electron reactions

**DOI:** 10.1093/nsr/nwaa075

**Published:** 2020-04-24

**Authors:** Yong-Xin Huang, Feng Wu, Ren-Jie Chen

**Affiliations:** Beijing Key Laboratory of Environmental Science and Engineering, School of Materials Science and Engineering, Beijing Institute of Technology, Beijing 100081, China; Advanced Technology Research Institute (Jinan), Beijing Institute of Technology, Jinan 250300, China; Beijing Key Laboratory of Environmental Science and Engineering, School of Materials Science and Engineering, Beijing Institute of Technology, Beijing 100081, China; Collaborative Innovation Center of Electric Vehicles in Beijing, Beijing 100081, China; Advanced Technology Research Institute (Jinan), Beijing Institute of Technology, Jinan 250300, China; Beijing Key Laboratory of Environmental Science and Engineering, School of Materials Science and Engineering, Beijing Institute of Technology, Beijing 100081, China; Collaborative Innovation Center of Electric Vehicles in Beijing, Beijing 100081, China; Advanced Technology Research Institute (Jinan), Beijing Institute of Technology, Jinan 250300, China

**Keywords:** multi-electron reactions, thermodynamic, kinetics, high-energy density, metal anodes

## Abstract

Multi-electron reaction can be regarded as an effective way of building high-energy systems (>500 W h kg^−1^). However, some confusions hinder the development of multi-electron mechanisms, such as clear concept, complex reaction, material design and electrolyte optimization and full-cell fabrication. Therefore, this review discusses the basic theories and application bottlenecks of multi-electron mechanisms from the view of thermodynamic and dynamic principles. In future, high-energy batteries, metal anodes and multi-electron cathodes are promising electrode materials with high theoretical capacity and high output voltage. While the primary issue for the multi-electron transfer process is sluggish kinetics, which may be caused by multiple ionic migration, large ionic radius, high reaction energy barrier, low electron conductivity, poor structural stability, etc., it is urgent that feasible and versatile modification methods are summarized and new inspiration proposed in order to break through kinetic constraints. Finally, the remaining challenges and future research directions are revealed in detail, involving the search for high-energy systems, compatibility of full cells, cost control, etc.

## DEFINITION OF MULTI-ELECTRON REACTION AND HIGH-ENERGY BATTERIES

With the development of electrochemical energy storage, more and more types of multi-electron reactions have been revealed based on the high-valence carriers, jumping valence change, broad electrochemical window, special anionic redox, etc. Although these reactions provide high energy density and application feasibility, the dynamic process during transfer and storage of ions should be improved to attain long cycle life and high rate performance. Moreover, the interfacial compatibility, structural stability, working conditions and safety of multi-electron reactions are also important issues. On the other hand, the high-performance metal anodes are conducive to building high-energy batteries, when used as counter electrodes for multi-electron electrodes. Hence, the dynamic optimization and stability promotion of high-energy batteries should take into account the multi-electron reaction and metal plating/stripping process.

In previous literatures, the understanding of multi-electron reaction has certain limitations and confusions, leading to slow development of high-energy systems [1,2]. Actually, the relationship between the multi-electron reaction and high-energy density is causal. The multi-electron reaction can be considered as an effective way for achieving high-energy systems, while the latter is a foremost goal for the former. The mass energy density of an electrode material (*E*_D_) can be calculated according to the extended Nernst equation:

(1) \begin{equation*} {E_D} = \frac{{{\Delta _r}{G^\theta }}}{{\Sigma {M_i}}} = - \frac{{nFE}}{{\Sigma {M_i}}}. \end{equation*}

Here, *n* refers to the number of the charge transferred during the per mole reaction, *F* is the Faraday constant, *E* represents the thermodynamic equilibrium voltage or the usual sense of electromotive force (emf) value, ∑*M_i_* is the sum of the mole weights or mole volumes of the reactants. The value of Gibbs formation energy of reactant can be calculated by the frequency property, which is attained through first principle calculations [3]. It can be confirmed that the high-energy density of the battery directly relates to the number of electrons involved in the reaction. Meanwhile, the high working potential and light mole weight are also important to achieve outstanding energy density. Therefore, the novel concept of multi-electron reaction has been proposed to construct next-generation energy storage devices based on high voltage and light element [1]. Recently, this meaningful scheme has been applied to conventional lithium-ion batteries (LIBs) and beyond LIBs, such as new charge carriers of Na^+^, K^+^, Mg^2+^, Al^3+^, etc. and especially chemical reactions involving anions (O^2−^, S^2−^) [4,5]. The Li-air (5 217 Wh kg^−1^) and Li-sulfur (2 567 Wh kg^−1^) are expected to achieve outstanding energy densities 2–10 times higher than those of the current LIBs (below 500 Wh kg^−1^) [6]. Based on the thermodynamic calculations of the energy densities for feasible battery systems and advanced storage materials, the theoretical energy density limits of these multi-electron reactions are significantly greater than those of the traditional single-electron reactions [7]. In particular, the full-cells assembled by the metallic lithium and conversion-/alloy-type electrodes with highly electronegative elements and light weights have the potential to be high-energy storage systems [8]. For instance, the Li-FeF_3_ and Li-FeS_2_ cells exhibit high energy densities of 1 643 and 1 295 Wh kg^−1^, respectively. Moreover, the Li-Li_1.25_Co_0.25_Mn_0.50_O_2_ cell delivers considerable energy density of 1 360 Wh kg^−1^ based on anionic redox reaction of Li-rich materials [9].

Theoretically speaking, the multi-electron concept can be regarded as a novel horizon for enhancing energy density of batteries. Hence, a thorough understanding of multi-electron mechanisms and electrochemical process is important to guide the design of superior electrode materials with highly redox activity and stable host structure. Moreover, the application of high-energy batteries based on the metal electrodes and multi-electron materials should take care of rate performance, safety matter, coulombic efficiency, environmental adaptability, etc. A series of advanced design techniques and optimization methods need to be adopted in subsequent studies.

## THERMODYNAMIC ANALYSIS OF MULTI-ELECTRON REACTION

### Analysis of thermodynamic formulas

The theoretical capacity and cycling stability limit can be speculated by the thermodynamic formula and redox reaction reversibility. Hence, the thermodynamic analysis not only reveals the types and mechanisms of multi-electron reaction, but also proposes the feasible electrode materials with ionic storage and stable host. On the whole, the multi-electron reactions are caused by more than one electron transfer. The general reaction formula can be of multi-electron storage described as:

(2) \begin{eqnarray*} {\rm{\alpha }}{A^{\beta + }} + \alpha \beta {e^ - } + \ \varepsilon \left( {{M_\theta }N} \right)\ \nonumber\\ \quad \to {A_{\left( {\alpha - \gamma } \right)}}{({M_{\theta - \delta }}N)_\varepsilon } + \ {A_\gamma }{M_{\delta \varepsilon }}.\end{eqnarray*}

Here, the *A* refers to the metal cations (such as Li^+^, Na^+^, K^+^, Mg^2+^, Ca^2+^, Zn^2+^, Al^3+^, etc.) or protons (H^+^ and NH_4_^+^); *e* represents the electron; *M* refers to the central metal cations with variable valence; *N* is the coordinated nonmetal anions with single- or poly-species, which may have valence change in redox reaction. In the condition of multi-electron reaction, the values of *α*, *ϵ*, *θ*, *δ* and *γ* coefficients are above zero and the product of *α* and *β* should be above one. It is noteworthy that the complex multi-electron reaction is not only caused by the valence change of central cations, but also may be ascribed to the redox process of coordinated anions or formation/breaking of chemical bonds [10,11]. In addition, the multi-electron concept can be extended to dual-ion batteries that can store cations and anions at the same voltage in two counter electrodes. According to the general reaction formula, the thermodynamic properties (Gibbs free energy, mass energy density (*E_M_*), volume energy density (*E_V_*), etc.) of the multi-electron process can be described as:

(3) \begin{eqnarray*} {\Delta _r\!}{G^\theta } &=& {\Delta _f} G_{{A_{\left( {\alpha - \gamma } \right){{({M_{\theta - \delta }}N)}_\varepsilon }}}}^\theta + {\Delta _f}G_{{A_\gamma }{M_{\delta \varepsilon }}}^\theta \nonumber\\ && -\, \left( {\alpha {\Delta _f}G_A^\theta + \varepsilon {\Delta _f}G_{{M_\theta }N}^\theta } \right),\end{eqnarray*}

(4) \begin{equation*} {E_M} = {\Delta _r\!} {G^\theta }\ \Big/\left(3.6\sum {\left( {\alpha {M_A} + \varepsilon {M_{{M_\theta }N}}} \right)}\right),\end{equation*}

(5) \begin{equation*}{E_V} = {\Delta _r} {G^\theta }\ \Big/\left(3.6\sum {\left( {\alpha {V_A} + \varepsilon {V_{{M_\theta }N}}} \right)}\right). \end{equation*}

For high-energy density batteries based on the metal anode, the voltage versus *A* metal at which an electrochemical element compounds with *A* can be calculated from the definition of the chemical potential:

(6) \begin{equation*} {\Delta _r}G\ = \ - nFV\ = {\rm{\ }}n\Delta \,{\mu _A} = {\rm{\ }}n\left( {{\mu _A} - \ {\mu _{{A^{\beta + }}}}} \right),\end{equation*}

(7) \begin{eqnarray*} V\ &=& {\rm{\ }} - \ \frac{{\Delta {\mu _A}}}{F} = \ - \ \frac{{{\rm{\Delta }}G}}{{\beta e{\rm{\Delta }}n}} \nonumber\\ &=& \ -\, \frac{{\Delta E + P\Delta V - T\Delta S}}{{\beta e\Delta n}} \nonumber\\ &\approx& -\, \frac{{\Delta E}}{{\beta e\Delta n}},\end{eqnarray*}

where *V*, *μ*, *e*, *G*, *n* and *E* are the voltage, chemical potential, absolute electron charge, Gibbs free energy, A^+^ number and internal energy, respectively. Because the values of entropy (*T*Δ*S*) and enthalpy (*P*Δ*V*) are several orders of magnitude smaller than the change in energy (Δ*E*), the contributions of the former can be neglected. Hence, the operating voltage as pivotal thermodynamic parameter of multi-electron reaction can be calculated based on the internal energies of reactants and products in general formula as:

(8) \begin{eqnarray*} V\ = {\rm{\ }} - \frac{{E({A_{\left( {\alpha - \gamma } \right)}}\left( {{M_{\theta - \delta }}N{)_\varepsilon }} \right) + E\!( {{A_\gamma }{M_{\delta \varepsilon }}} ) - \varepsilon E\!( {{M_\theta }N} ) - \alpha E\!( A )}}{{\alpha \beta e}}.\hphantom{00000000000000000000000} \end{eqnarray*}

The most widespread approach for obtaining total energy of different compounds is the density functional theory (DFT) method. In the following, the detailed multiple electron transfer processes originating from chemical reactions between *A*, *M* and *N* components are described one by one.

### Different kinds of multi-electron reactions

**Figure 1.**
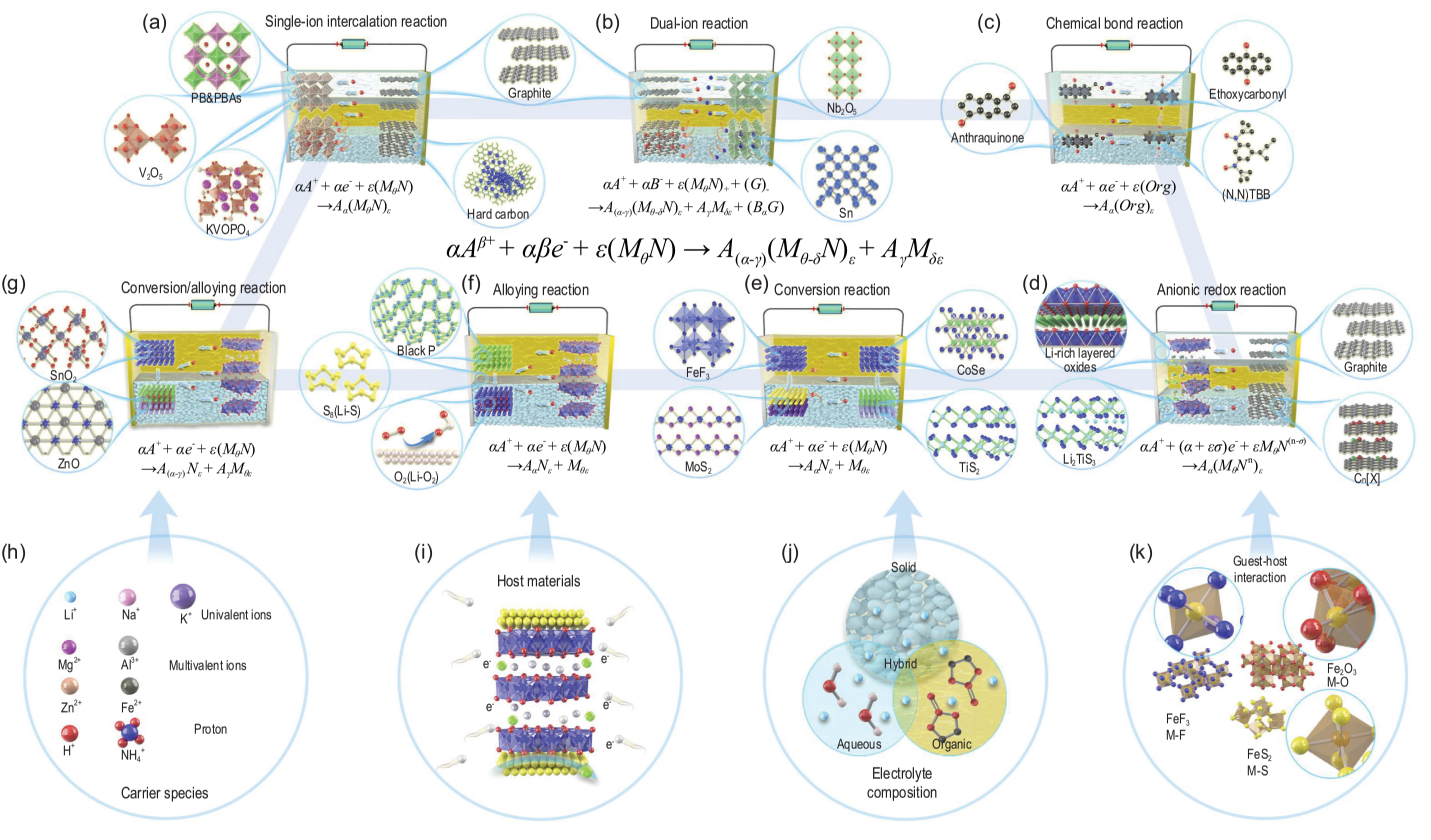
Schematic illustration of different kinds of multi-electron reaction mechanisms and the corresponding electrode materials. (a) Single-ion intercalation reaction. (b) Dual-ion reaction. (c) Chemical bond reaction. (d) Anionic redox reaction. (e) Conversion reaction. (f) Alloying reaction. (g) Conversion-alloying reaction. Four key factors for optimizing multi-electron reaction: (h) carrier species; (i) host materials; (j) electrolyte compositions; (k) guest-host interaction.

On the basis of different reaction paths, the multi-electron reactions can be divided into seven types (Fig. 1). Moreover, the representative electrode materials for different reaction types are presented in Fig. 1 to explain the multi-electron process in detail. These multi-electron reactions are closely related to the working condition, including voltage window, electrolyte property, current density, etc. [12]. Thus, whether it is a multi-electron reaction or high-energy electrode, the concrete reaction mechanism and the corresponding condition should be pointed out simultaneously.

First, the single-ion intercalation reaction with multi-electron transfer indicates the phase transition process in cation-rich materials or high-valence cations storage in the corresponding compounds. In the former, the reactants transform from the initial phase to another phase with more than one mole cation storage or release, such as Prussian blue (from cubic to rhombohedral phase) [13], Li_x_V_2_O_5_ (from α-phase to ω-phase) [14], Li_x_VOPO_4_ (from ϵ-phase to β-phase) [15], etc. The phase transition process can be ascribed to the increase of active sites and deformation of lattice structure. In another case, the bivalent or trivalent cations are inserted into layered or three-dimensional (3D) frame structures with more than 0.5 or 0.33 mole cations storage or release, such as layered oxides (Mg_x_MnO_2_) [16], Prussian blue analogues (Ni_3_Fe(CN)_6_)_2_ [17], and polyanionic compounds (MgMnSiO_4_) [18]. These electrodes usually exhibit higher standard potential with enhanced safety and smaller cation size with enabled intercalation dynamics.

Second, the dual-ion reaction suggests that the cations and anions in electrolytes are stored into cathode and anode simultaneously during the charging or discharging process [19]. When more than 0.5 mole ions are stored in each electrode, the multi-electron reaction in dual-ion systems can provide outstanding energy density. For dual-ion systems, the key property of multi-electron electrodes is the capability of storing both cation and anion. Typically, environmentally friendly and stable graphite [20], flexible and low cost organic materials [21], high-voltage layered metal oxides [22] and high-active metal-organic frameworks [23] are used as feasible electrode materials with the ability of anion intercalation. In order to achieve multi-electron reaction, the high operating voltage (>4.5 V) and open host structure should be designed to accommodate PF_6_^−^, TFSI^−^, ClO_4_^−^ and AlCl_4_^−^ with large radius and high electronegativity. Therefore, the components and properties of electrolytes are very important to dual-ion systems, especially for species and concentrations of anions [24].

Third, multi-electron storage also can be realized in organic compounds with carbonyl (C = O) or free radical (N^+^) functional groups [11]. The organic free radical compounds refer to aliphatic or non-conjugated polymers, which hold rapid electron transfer capability and high chemical stability. In particular, the organic electrode materials usually have light weight and flexible molecular segments, which exhibit high weight energy density and enough space for tolerating cations with large ionic radius. Furthermore, the thermodynamic reaction mechanism and dynamic reaction rate of organic materials are different from that of generally inorganic electrodes, which may provide moderate voltage and enhanced rate performance. In organic electrodes, the voltage profiles are plateau type, which can be regarded as a kind of first-order phase transition reaction with low voltage polarization [11]. Currently, the representative organic electrode materials contain carbonyl compounds (anthraquinone and benzoquinone) [25], free radical compounds (chalcogenoviologen) [26] and their derivatives, which can deliver high capacities above 250 mAh g^−1^ with storage of cations in multiple active sites.

Fourth, the anionic redox reaction mainly means that the anions in layered transition metal oxide cathodes can get and lose electrons during the redox process of cations, which can be attributed to unusual band structures with non-bonding oxygen states [27]. Generally, the orbital overlaps between *d* orbitals of transition metal and *p* orbitals of oxygen, form bonding (M-O) and antibonding (M-O)^*^ bands [28]. The single cation redox reaction is caused by the electron donor effect of antibonding (M-O)^*^ bands and structural stability effect of bonding (M-O) bands. In some special lithium-rich materials, the small overlaps between *p* orbitals of O and *s* orbitals of excess Li lead to a weak bonding (Li-O) band, inducing a new energy level above the bonding (M-O) band and providing excess electrons. The essence of this special mechanism is existence of lone-pair oxygen or unhybridized O *2p* state [29]. In addition, the relative positions of O *2p* non-bonding and (M-O)^*^ antibonding bands is another interfering factor for realizing reversible anionic redox. Highly covalent M-O bonding is beneficial to the redox reaction of anions. Although these mechanisms are worth further discussion, the typical anionic redox cathodes have been developed to attain high-energy density, involving Li-rich oxides [27], Li_2_TiS_3_ [30], Na_3_RuO_4_, etc. [31]. Recently, a novel anionic redox reaction of halogen ions has been investigated in aqueous Li-ion batteries with graphite electrodes [32]. The highly reversible capacity of 243 mAh g^−1^ and multi-electron transfer process can be observed in the voltage range of 4.0–4.5 V.

Fifth, the conversion reaction is a kind of phase transition reaction with the formation of two or more new species during the cation storage process. Due to the complete utilization of highly active transition metals, the electrode materials based on the conversion reaction can provide high theoretical capacity and broad operating voltage range. This kind of reversible cation storage can be observed in transition metal fluorides (FeF_3_) [33], sulfides (MoS_2_) [34], selenides (CoSe) [35], phosphides (CoP), etc. [36], which are used as both cathode and anode materials. It is worth noting that multi-electron transfer in conversion reaction is not a one-step process, and should be divided into multiple steps with different mechanisms. For instance, lithiated processes of FeF_3_ contain two steps: first, lithium ions are inserted into the interlayer of FeF_3_ through one electron transfer; second, two extra lithium ions and LiFeF_3_ precursor are transformed into LiF and metallic Fe corresponding to the conversion mechanism.

Sixth, the alloying reaction can be interpreted as a phase integration process between cations and alloyed anodes with multi-electron transfer. The result of the alloying reaction is in favor of high-energy density owing to the multi-ion storage and low operating potential. Group IVA and VA elements (such as Si, P, Sn, Sb, Ge, As, etc.) are able to form binary intermetallic compounds with different cations, which is determined by the redox potentials of alloyed anode versus cation/metal [37]. For example, significant polarization and rapid capacity fading are observed in alloying reactions between Si and Na^+^ induced by the similar redox potentials of the alloying process and Na plating process [38]. In the same vein, a number of alloying reactions will be terminated before they reach the final state. Fortunately, this problem can be solved by nanotechnology or bimetallic compounds, which may build a new phase diagram [39]. In a broad sense, metal-S and metal-O_2_ batteries are also alloying reactions forming M_x_S and M_x_O products, corresponding to high theoretical energy density above 1000 Wh kg^−1^ [4]. However, the cation storage processes in these two batteries are more complex than general alloying electrodes, and they can be identified as a multiple reaction with different numbers of electron transfer. According to the DFT results, the primary three-step reaction in Li-S batteries can be described as: S_8_ ↔ S_4_^2−^ ↔ S_2_^2−^ ↔ S^2−^, resulting in an ambiguous thermodynamic process [40]. Similarly, the Li-O_2_ battery in non-aqueous electrolytes involves two-electron and four-electron transfer pathways.

Seventh, the combination of conversion and alloying reaction is regarded as a new multi-electron transfer mechanism. The representative materials are composed of active IVA or VA elements and coordinated VIA elements. Moreover, zinc element also can participate in the alloying reaction with Li^+^ and Na^+^ [41]. Typically, the metal oxides with conversion-alloying mechanism in lithium ion batteries are firstly reduced to the elemental metal and Li_2_O. Subsequently, the alloying reaction between the metal and lithium will take place at low potential. It should be emphasized that the irreversible or partially reversible conversion reaction may lead to remarkable capacity loss, which is ascribed to the poor conductivity of metal oxides. According to the number of metal elements, the conversion-alloying electrodes can be divided into single-metal compounds (i.e. SnO_2_) and multi-metal compounds (i.e. Zn_2_SnO_4_ and CoSnO_3_) [42–44] while the multi-metal compounds can contain one or more active metal elements.

In brief, there are seven kinds of multi-electron reaction mechanisms based on the different active elements and cation storage paths. These multi-electron electrodes generally deliver higher energy density than that of commercial lithium-ion batteries. An advanced rechargeable battery using multi-electron cathodes (i.e. polyanionic cation-rich compounds or layered cation-rich oxides) and multi-electron anodes (i.e. silicon or metal oxides) can achieve high energy density above 350 Wh kg^−1^. If the metal anodes are applied to the next-generation batteries, the practical energy density of multi-electron batteries will exceed 500 Wh kg^−1^ [45]. The development of Li-S and Li-O_2_ batteries is to be expected due to the ultrahigh upper limit of energy density. Although the reaction types, theoretical energy densities and theoretical voltages of multi-electron processes have been revealed by the theoretical calculations and analysis, intensive research should be carried out on specific electrode materials to understand a variety of multi-electron storage pathways. As shown in Fig. 1, the host structure, guest-host interaction, anionic redox and electrolyte species of these electrode materials are important factors for multi-electron processes. Recently, more and more novel cations and host materials are employed to build multi-electron systems, such as Fe-S batteries (S_8_ ↔ FeS_2_ ↔ Fe_3_S_4_ ↔ FeS) [46], Cu-S batteries (S_8_ ↔ CuS ↔ Cu_2_S) [47], Na-layered Na-rich oxide batteries (O^2−^ ↔ O_2_^2−^) [48], dual-carbon batteries (K^+^/PF_6_^−^ ↔ K_x_C/[PF_6_]_x_C), etc. [49]. These multi-electron reactions usually operate at high voltage even close to 5 V and the instability of electrodes and electrolytes is an important issue, which puts forward higher requirements for electrolytes. Hence, the aqueous electrolyte cannot be applied to conversion and alloy reaction. Therefore, solid and hybrid electrolytes receive more attention with regard to enhancing safety and compatibility. Moreover, the interactions between cations and host structures lead to different reaction degrees, corresponding to different numbers of transferred electrons [33]. The multi-electron reactions with various thermodynamic properties based on seven reaction types will be continuously developed to meet the needs of high capacity, abundant resources and low cost.

### Different types of multi-electron materials

**Figure 2.**
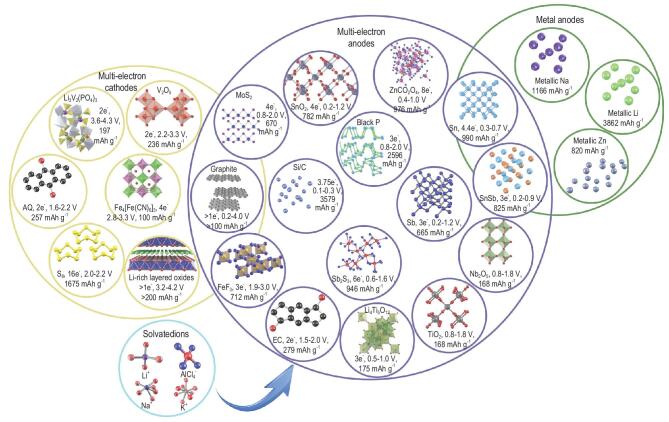
Theoretical specific gravimetric capacities, redox potentials, molar electron transfer numbers and chemical structures of promising multi-electron host materials for high-energy density batteries, which are discussed based on the lithium metal counter electrode. The representative metal anodes can be applied to high-energy density batteries. The structures of solvated cations are shown in the inset, which is an important factor for multi-ion and high-valence cations storage.

As shown in Fig. 2, the potential multi-electron materials and metal electrodes are generalized to speculate the reasonable design of high-energy density batteries. To achieve the high energy density, the multi-electron reactions occurring in cathodes should hold high redox voltages and high coulombic efficiencies. Therefore, the polyanion compounds, Li-rich oxides and metal-organic frameworks (MOFs) are feasible cathodes for multi-electron reactions. Owing to the strong electronegativity and high inductive effect of polyanions, Li_3_V_2_(PO_4_)_3_ exhibits extremely high redox potentials of 3.6–4.3 V and high theoretical capacity of 197 mAh g^−1^ with two-electron transfer [50]. The initial charging curve of monoclinic Li_3_V_2_(PO_4_)_3_ presents multi-platform (3.6, 3.7 and 4.1 V *vs* Li/Li^+^) corresponding to the step by step multi-electron transfer process. The first two platforms at 3.6 and 3.7 V are assigned to 0.5 Li^+^ extraction, resulting in the formation of intermediate ordered phase Li_2.5_V_2_(PO_4_)_3_. Subsequently, one Li^+^ extraction at 4.1 V indicates a two-phase process of Li_2_V_2_(PO_4_)_3_ → LiV_2_(PO_4_)_3_ with full oxidation of V^3+^ to V^4+^. The anionic redox in Li-rich layered oxides can provide outstanding capacity (>200 mAh g^−1^) and high operating voltage of 3.2–4.2 V, illustrating the superior application prospect to high-energy batteries [28,30]. On the basis of the solid solution phase, the rocksalt Li_2_MO_3_ (M = Ti, Ru, Mn, Mo, Sn, etc.) family materials can deliver one broad platform during the charging process, involving metal cationic (Mn^+^ → M^(n+1)+^) and anionic (O^2−^ → O_2_^2−^) reversible redox processes. To improve the thermodynamic stability and coulombic efficiency of these Li-rich layered oxides, the partial M element is replaced by the element that is not easily reduced [51]. Prussian blues and their analogues (PB and PBAs) are pursued as multi-electron cathodes for inserting a variety of cations (i.e. Li^+^, Na^+^, K^+^, Mg^2+^, Al^3+^, etc.) [17,52]. Because there are dual-redox active centers in framework structures, the PB and PBAs electrodes can exhibit considerable capacity above 120 mAh g^−1^ and one charging platform at 3.0 V, corresponding to the phase transformation between cation-rich rhombohedral phase and cation-poor cubic phase [13]. Furthermore, cation-poor organic materials, V_2_O_5_, FeF_3_, S and O_2_ electrodes can be utilized in metal batteries to achieve multi-electron reactions. Especially with Li-S and Li-O_2_ batteries, they have the potential to reach practical energy density above 600 Wh kg^−1^ in the next few years. However, the complex redox mechanisms of these two batteries need to be further investigated to reveal the electron transfer process, discharge intermediates, electrode/electrolyte interaction, etc.

There are more kinds of multi-electron materials that can be selected as advanced anodes for high-energy density batteries. The cation-rich Li_4_Ti_5_O_12_ shows feasible theoretical capacity of 175 mAh g^−1^ in the voltage range of 1.0–2.5 V with three-electron transfer [53]. When the fully discharged voltage is reduced to 0.01 V, the Li_4_Ti_5_O_12_ delivers high capacity of 293 mAh g^−1^ with five-electron transfer. The Nb_2_O_5_ is another multi-electron anode based on the phase transformation reaction [54]. It can display larger theoretical capacity of 200 mAh g^−1^ and one voltage platform around 1.2–1.6 V, corresponding to formation of Li_2_Nb_2_O_5_. Hence, the energy density of intercalation reaction is relatively limited due to the incomplete utilization of high valence cations. As a common conversion reaction electrode, MoS_2_ electrode exhibits great capability to store Li^+^, Na^+^, K^+^ and Mg^2+^ with four-electron transfer [55]. In Li-ion batteries, it shows a high theoretical capacity of 670 mAh g^−1^ and low redox potential of 0.8–2.0 V, indicating that it can be used as a cathode in metal batteries. The graphite-like structure of MoS_2_ provides advantageous electrochemical activity and cycling stability. The conversional FeF_3_ also can achieve excellent performances both as cathode and anode, which is worth thoroughly investigating in next-generation batteries [33]. To obtain ultrahigh capacity, alloyed anodes have been developed including metalloids, metals and bi-metal compounds [37]. The metalloid Si and black P with light weights can deliver extremely high theoretical capacity of 3 579 and 2 596 mA h g^−1^ corresponding to 3.75 and 3 electrons transfer, respectively. The redox potentials approaching to Li/Li^+^ couple lead to the extensive applicability and high energy density of Si and P anodes. The metal anodes usually have good conductivity and high reaction activity. For instance, the metallic Sb shows high lithium storage capability of 665 mAh g^−1^ with three-electron transfer in the voltage range of 0.2–1.2 V. Similarly, the alloy binary compound SnSb provides outstanding capacity of 825 mAh g^−1^ contributed by the two-step redox reaction of double active elements [56]. It should be noted that the Li_3_Sb formed in the first step are rearranged in Sn particles, which buffer the volume expansion and enhance the reaction kinetic of lithiated Sn. Currently, conversion-alloying reactions have attracted great attention since their complex compositions provide different electrochemical characteristics [43]. Meanwhile, the controllable structure and varied chemical composition (oxides, sulfides, selenides, phosphides and multiple metal elements) are important advantages in designing excellent thermodynamic and kinetic properties [57]. Moreover, the organic compounds and graphite are available choices for high-energy anodes. Although graphite cannot achieve multi-electron reaction based on the general lithium-ion battery, it can be applied to both cathode and anode for dual-ion batteries with a high number of transferred electrons.

Recently, metal anodes have attracted wide attention due to high-energy densities and preferred redox potentials. The metallic Li, Na, K, Mg, Al and Zn anodes not only deliver ultrahigh capacity but also present low redox potential. Especially, Li metal is regarded as the ‘Holy grail’ of high-energy density anodes because it has the lowest redox potential of −3.040 V *vs* NHE and the highest theoretical capacity of 3860 mAh g^−1^. Although Na and K metals exhibit similar chemical and electrochemical properties with Li metal, there is still a big gap in their thermodynamic and kinetic properties. It is an established fact that other metal anodes show sustainability and low cost beyond Li metal, indicating the necessity of developing different metal anodes. Owing to the high melting points and dendrite-free properties, the Mg metal may become a good candidate for multi-electron systems matched with MgMnSiO_4_ or PB cathodes [18]. Moreover, Zn metal is also adequately exploited to optimize alkaline zinc-manganese dioxide batteries. Therefore, the multi-electron cathodes and metals anodes are regarded as superior candidates for high-energy density systems in the future.

The electrolyte in multi-electron batteries usually plays an important role in realizing insertion of high-valence cations and stability of dual-ion batteries. In other words, the thermodynamic properties of multi-electron batteries are influenced by the electrolytes. For example, the revolution of aluminum-metal batteries is caused by the novel AlCl_3_/1-ethyl-3-methylimidazolium chloride (EMICl) electrolyte, which can form AlCl_4_^−^ inserted into an interlayer of graphite [58]. As is well known, dual-ion batteries with high operating voltage have difficulty attaining stable cycles and sufficient coulombic efficiency. The development of high-concentration electrolytes and various additives becomes a vital approach for improving dual-ion systems [59]. Owing to complex multi-electron transfer processes and intermediate products, there are many requirements for the electrolytes in Li-S and Li-O_2_ batteries, such as low solubility, good wettability and uniform film formation [60]. Thus, the critical impact of electrolytes, interfaces and solvated ions in multi-electron systems should be deeply investigated, especially for compatibility with different cations.

## KINETIC ANALYSIS OF MULTI-ELECTRON REACTION

### Kinetic process of multi-electron reaction in metal batteries

**Figure 3.**
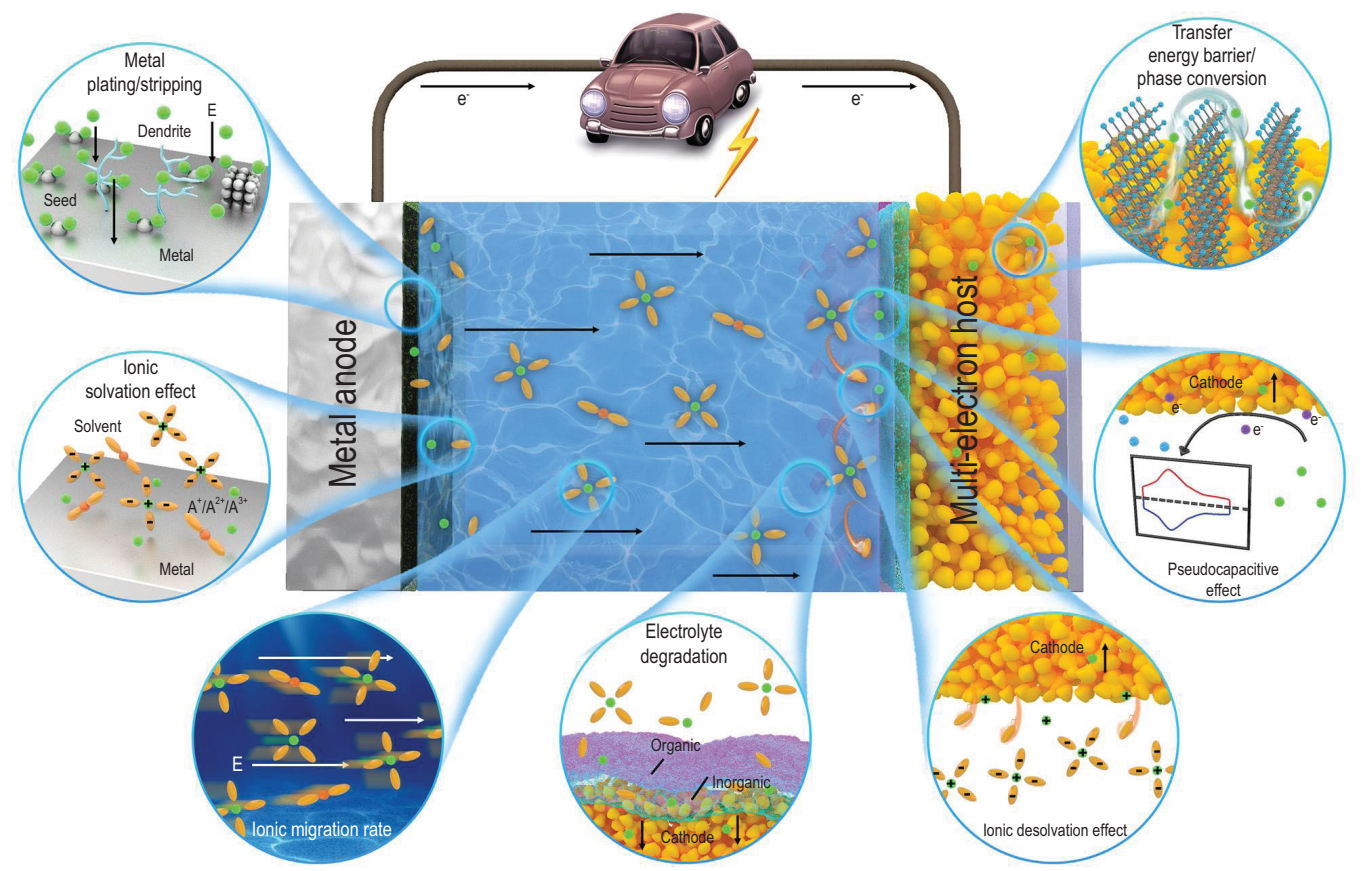
Schematic illustration of complex kinetic processes during ionic transfer and storage in the general multi-electron system with high-energy density, involving metal plating/stripping, ionic solvation effect, ionic migration rate, electrolyte degradation, ionic de-solvation effect, pseudocapacitive effect, ionic diffusion in electrode bulk and phase conversion processes.

In the high-energy density full-cells assembled by multi-electron cathodes and metal anodes, the kinetic process of cation transfer has been described in Fig. 3, which is divided into seven steps. Versatile kinetic processes are covered in this representative system, involving cation transfer in a multi-electron cathode, metal anode, electrolyte and interface. During the charge-discharge process, the plating or stripping of a metal anode is dominated by the nucleating and growth dynamics [61]. Typically, the different nucleation mechanisms are observed in Li and Mg metals, which can be ascribed to the stronger binding energy of Mg-Mg bonds and higher free energies of 1D growth for Mg metal [62]. The rapid ion diffusion on a Mg electrode results in a tendency to deposit toward the surrounding area rather than gathering in one place. In contrast, the Li and Na metal with low surface energy and a high diffusion barrier easily form dendrites. From the view of kinetics, the flat surfaces, affinity sites and low overpotentials may contribute to the uniform growth of Li metal. The kinetics of nucleation and growth processes of metal anodes is widely described by the ‘Sand time’ model [63]. Sand time is the time for ionic concentration going to zero near the electrode. Meanwhile, a lot of metal ions are adsorbed to the electrode surface due to the strong negative electric field, and then the dendrite begins to form during the fast charge-discharge. The Sand time τ of metal anodes in multi-electron batteries (i.e. Li^+^, Na^+^, Zn^2+^, Cu^2+^, etc.) can be calculated by the following formulas [64]:

(9) \begin{equation*}\tau \ = {\rm{\ \pi }}D{\left[\frac{{{C_0}e{Z_\beta }\left( {{t_a} + {t_c}} \right)}}{{2J{t_a}}}\right]^2},\end{equation*}

(10) \begin{equation*}D\ = \frac{{{t_a}{D_c} + {t_c}{D_a}}}{{{t_a} + {t_c}}},\ \end{equation*}

where *D* is the diffusion coefficient, *C*_0_ is the initial cation concentration, *e* is the electronic charge, *Z*_β_ is the cationic charge number, *J* is the current density, *t*_a_ and *t*_c_ are cationic and anionic migration numbers, and *D*_a_ and *D*_c_ are cationic and anionic diffusion coefficients. However, the Sand time model needs to be revised at low current density [65]. The nucleation time is inversely proportional to the quadratic power of the current density, while the short circuit time is inversely proportional to the current density.

After stripping from the metal anodes, the solvation effect between cations and solvent molecules is a critical parameter for ionic transfer and reaction kinetics. Correspondingly, the de-solvation process can be observed at the surfaces of multi-electron electrodes, which is an important step for interfacial cation transfer. The low solvation and de-solvation energies of cations lead to the faster ion transfer rate in interfaces. The solvation effect is affected by the cation species, solvent species and electrolyte concentration [66]. Taking these factors into account, the solvated structures and de-solvation energies of cations can be built and calculated by the following formula:

(11) \begin{equation*}{E_{\mathit{de-solvation}}} = {E_{\mathit{cations}}}\ + {E_{\mathit{solvent}}} - {E_{\mathit{complex}}}.\end{equation*}

According to the calculation results, the de-solvation energies show a remarkable increasing tendency in the order of Na^+^ < Li^+^ < Mg^2+^, indicating that the weaker Lewis acidity and lesser positive charge number are beneficial to the low de-solvation energy [67]. Moreover, the de-solvation energy shows a linear correlation with the electrostatic and polarization contributions of different solvents. The ether-based electrolytes usually deliver lower de-solvation energies compared with that of carbonate-based and sulfolane-based electrolytes. The electrolyte concentrations mainly influence the coordination numbers and structures of cations, leading to different de-solvation energies.

The migration dynamics of cations in electrolytes also have a certain impact on the dynamic performances of full-cells, which are reflected by the important parameter of ionic conductivity. Typically, the ionic conductivities of liquid organic electrolytes can be described as the following formula:

(12) \begin{equation*}{\rm{K\ }} = \mathop \sum \limits_i \frac{{{{({Z_i})}^2}F{C_i}}}{{6\pi \eta {r_i}}},\ \end{equation*}

where *Z*_*i*_ and *C*_*i*_ are the charge number and molar concentration of *i* ion, respectively. *F* is the Faraday constant, *η* is the viscosity of the electrolyte and *r*_i_ is the solvation radius of *i* ion. Hence, the solvation structures, electrolyte viscosities and cation species need to be optimized to attain high ionic conductivities. For univalent cations in ester-based electrolytes, the weaker-solvated Na^+^ and K^+^ ions exhibit a superior transfer dynamic than Li^+^ ions [68]. According to this theory, the divalent cations usually show stronger solvation energies and lower ionic conductivities compared with the univalent cations.

Generally, the interface transfer dynamics of cations is the determining factor of reaction rate, corresponding to the ionic transfer in solid electrolyte interface (SEI). In any event, the cation diffusion coefficient in the SEI films is much lower than that in the liquid electrolyte. Although the formation processes and chemical compositions of SEI films in metal anodes and multi-electron electrodes are different, the ionic diffusion pathways in these SEI films are similar. The ion transfer in SEI films follows the ‘two layer mechanism’ [69]. The cations diffusion in a porous organic layer is the pore diffusion mechanism, while the cations diffusion in a dense inorganic layer is the interstitial knock-off. These two mechanisms may contribute to the cations transfer in a mixed region together. However, some researches confirm that the cations transfer in SEI films is mainly liquid diffusion through porous structure rather than solid diffusion [70]. The complex interfacial issues should be further studied in the next step. According to the results of electrochemical impedance spectroscopy (EIS), the diffusion coefficients of cations in the SEI films can be calculated from the following equation [71]:

(13) \begin{equation*} D\ = {\rm{\ }}0.5{\left(\frac{{RT}}{{A{n^2}{F^2}{\sigma _\omega }C}}\right)^2}, \end{equation*}

where *R* is the gas constant, *T* is the temperature, *A* is the area of electrode surface, *n* is the number of electrons per molecule during reduction, *σ*_ω_ is the Warburg factor and *C* is the cation concentration. It can be inferred that the high electron number and cation concentration in SEI films may lead to low cation diffusion coefficient.

In the bulk of multi-electron electrodes, the diffusion energy barrier of cations and formation/break energy of chemical bonds are the main dynamic parameters. In intercalation reaction, the guest-host interaction between the cations and electrodes is a primary factor for transfer dynamics, while the formation energy is another impact factor. The smaller cation sizes and broader migration paths are conducive to the fast transfer of cations in lattice. Moreover, the electronic and ionic configurations play a critical role to cations transfer in a host structure [72]. Typically, the special, fast ionic conductor with low energy barriers in gap positions can lead to jumping-type migration of cations in adjacent sites [73]. Furthermore, the migration rate of cations is remarkably increased from 1D to 3D migration pathways. For phase transformation reaction, the weak guest-host interaction in electrodes and stable formation phase result in lower formation energy. For instance, the chemical banding energy of M-Se bond is weaker than that of M-S bond and M-O bond since the high electronegativity of O^2−^ and S^2−^ causes a strong inductive effect to form a firm connection in the host structure [74]. Thus, the conversion reaction of metal selenides holds a significant dynamic advantage. Meanwhile, the low electrical conductivity is also an important limiting factor for kinetic processing of metal compounds. For alloying reaction, the bond energies between cations and alloyed anodes determine the kinetic performance, which usually increases with the ionic storage [75]. The kinetic performances of Li-S and Li-O_2_ batteries are limited by the low electrical conductivities and poor wettability of electrode/electrolyte interfaces. Furthermore, the formation and fracture of Li-S and Li-O bonds show certain irreversibility caused by the low reaction activity. Fortunately, the lithiated/delithiated reaction kinetics in Li-S and Li-O_2_ batteries can be promoted by the catalytic assistants who reduce the formation energy between Li-S and Li-O_2_ [6]. Similarly, for anionic redox reaction, the dominant factor for kinetic performance is formation and fracture of Li-O and Li-S bonds instead of the diffusion barrier of cations in layered transition metal oxides.

The volume expansion and grain pulverization of electrodes exhibit a direct association with the reaction kinetics, especially for multi-electron reactions. For example, the relative volume change ranges for Li-based and Na-based conversion reactions are concentrated in 100%–200% and 200%–300%, respectively [76]. Generally speaking, the volume expansion ratios of different conversion anodes exhibit an increasing tendency in the order of halogens <oxides, sulfides <nitrides, phosphides. Therefore, the volume expansion degrees of multi-electron electrodes are influenced by the cation species and multi-electron materials, which can be further optimized by constructing a rapidly ionic and electrical transfer structure.

In addition to the host materials, the redox reactions that occur on the electrode surface are an important means of cation storage. The pseudocapacitance process is defined as a Faraday process on the surface of electrodes based on the ionic adsorption and desorption. The pseudocapacitance behavior is determined by the material structures and carrier species [77]. For instance, the significant pseudocapacitance effect can be attained by the nano-sized multi-electron materials. Pseudocapacitance behavior lies between capacitance behavior and battery behavior, which exhibits broad redox peaks during the charge-discharge process without specific voltage platforms. The pseudocapacitance reaction can be captured in both organic and aqueous systems, while multi-electron materials with intercalation, conversion and alloying reactions can be used as electrodes [78]. According to the cyclic voltammetry (CV) curves measured at different rates, the contribution of response current to total current can be quantitatively calculated by the following formula [79]:

(14) \begin{equation*}i = {k_1}v + {k_2}{v^{1/2}},\end{equation*}

where *i* is total current and *k*_1_$v$ and *k*_2_$v$^1/2^ correspond to the current contributions from capacitance behavior and diffusion behavior, respectively. The pseudocapacitance contributions may increase with the rising current densities. Generally speaking, the capacitance-dominant processes concentrate on the non-platform regions, while the diffusion-dominant processes are located at platform position. The pseudocapacitance reaction presents advanced cation storage kinetics and considerable capacity since the redox reaction takes place on the electrode surface without a diffusion barrier.

### Synergistic diffusion effect in multi-electron electrodes

The superior diffusion dynamics of cations in multi-electron electrodes is the key point for great reaction activity and stability of high-energy density batteries. The diffusion barriers of cations dominate the rate of multi-electron reactions, which is also the research emphasis of this review. The diffusion process is an essential step in the multi-electron reaction, whether it is intercalation, conversion or alloying reaction. According to the above-mentioned concept, the diffusion dynamics of monovalent Li^+^ and bivalent Mg^2+^ ions in multi-electron electrodes with different reaction mechanisms have been investigated by the first principle calculation [80]. The representative intercalation materials (Li_3_V_2_(PO_4_)_3_ and FeFe(CN)_6_), conversion material (MoS_2_) and alloying material (sulfur) are treated as host materials for holding migration of these two ions. The target of theoretical simulation is to reveal the dynamics characteristics of various kinds and different amounts of cations during the diffusion process in multi-electron electrodes.

**Figure 4.**
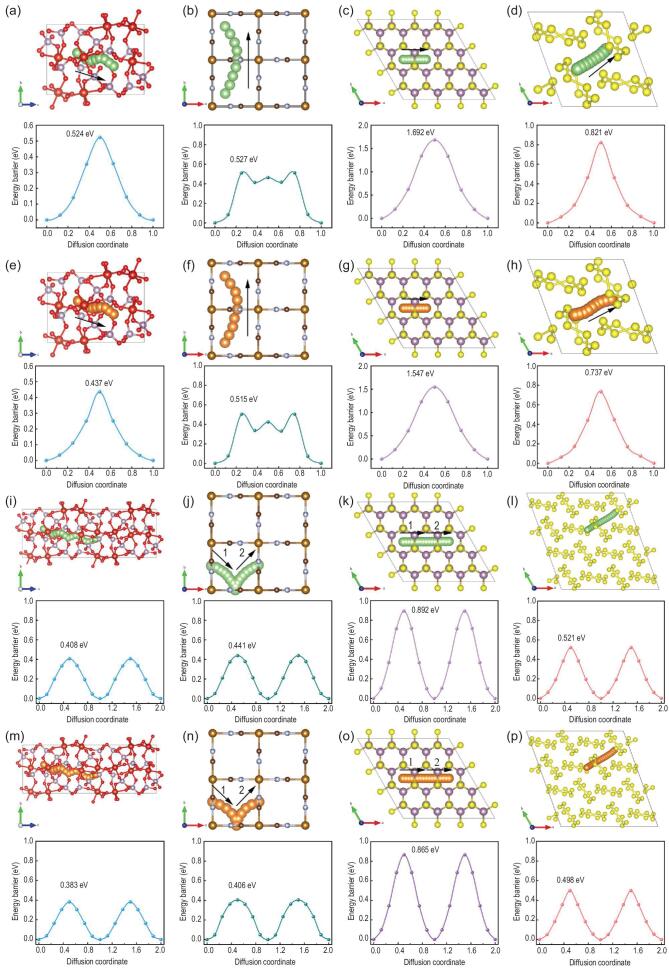
DFT calculation for different migration energy barriers of representative multi-electron materials, involving Li_3_V_2_(PO_4_)_3_, FeFe(CN)_6_, MoS_2_ and sulfur electrodes. (a–d) Front views of possible single Li^+^ migration paths between adjacent storage sites. (e–h) Front views of possible double Li^+^ migration paths between adjacent storage sites. (i–l) Front views of possible single Mg^2+^ migration paths between adjacent storage sites. (m–p) Front views of possible double Mg^2+^ migration paths between adjacent storage sites. The corresponding migration energy barriers of the above paths are shown under them.

As shown in Fig. 4, the Li^+^ and Mg^2+^ ions diffuse between two adjacent cations storage sites along the pathways with lower energy barriers. For instance, the Li^+^ and Mg^2+^ ions migrate in NASICON-type Li_3_V_2_(PO_4_)_3_ and layered MoS_2_ with 3D and 2D transfer pathways, respectively, which can be regarded as an important reason for the difference of diffusion barriers. It can be speculated from the diffusion barriers of Li^+^ and Mg^2+^ ions in different multi-electron electrodes that the open 3D transfer pathways and highly electrical conductivities are conducive to the rapid transfer of cations [81]. In particular, the fast ionic conductor delivers a low cation diffusion barrier (∼0.437 eV) due to the abundant lattice defects and special rigid structure. For different cations, the diffusion barriers of Mg^2+^ in these multi-electron materials are similar or lower than that of Li^+^, which can be ascribed to the similar ionic radius and weaker binding energies of Mg^2+^ [82]. This phenomenon can be observed in other multi-electron reactions based on the various cations (Na^+^, K^+^, etc.), illustrating that this mechanism can be extended to other systems [83]. Therefore, the multi-electron reactions also can realize superior kinetics in some specific materials with appropriate ion channels and weak binding energies. It is noteworthy that Li^+^ and Mg^2+^ ions in sulfur electrodes exhibit significant differences in transfer pathways, which may be caused by interaction between cations and host materials.

Furthermore, the multi-electron reactions with cooperative Li^+^ or Mg^2+^ diffusion have been investigated to understand the origin of the fast cation transfer in some specific materials. For convenient research, the double ions transfer in multi-electron electrodes is considered a research target, showing similar and connected migration pathways for single ions. Both Li^+^ and Mg^2+^ ions exhibit an enabling effect for low energy barriers during the cooperative migration process in Li_3_V_2_(PO_4_)_3_, FeFe(CN)_6_ and MoS_2_ which can be attributed to the strong coulomb interactions between mobile cations. In fact, the cations located at high-energy and low-energy sites migrate simultaneously, providing a canceling effect of different motion directions [84]. This phenomenon was first found in NASICON-type materials, and then it was applied to various cations and host materials, including layered and framework structures [85]. The quantity and uniformity of cations in crystal structures are important factors for cooperative migration effect. Furthermore, the cooperative migration effect of Mg^2+^ ions is stronger than that of Li^+^ ions due to the weaker interactions between Mg^2+^ ions and polarizable anions. However, this positive effect is difficult to capture in materials with isotropic migration pathways, which deliver high symmetry and equivalent pathways [86]. Therefore, the NASICON-type materials with anisotropic ion channels can exhibit significant differences in single-ion and double-ion transfers. The metal ion doping and heterostructure building are feasible means to change the transfer pathways, leading to low diffusion barriers.

### Advanced strategies for improving kinetic properties

**Figure 5.**
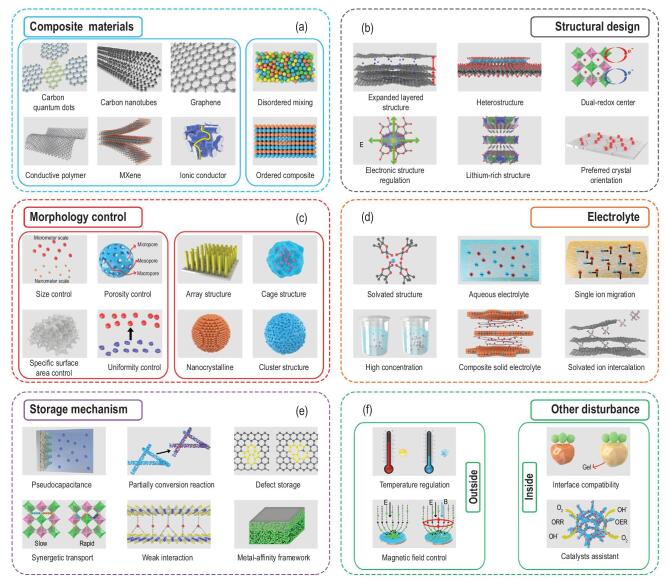
Summary of advanced materials, mechanisms and methods for constructing high-energy density batteries via improved multi-electron reactions and enhanced kinetic process. (a) Composite materials. (b) Structural design. (c) Morphology control. (d) Electrolyte. (e) Storage mechanism. (f) External disturbance.

There is immense potential to optimize the reaction kinetics—as opposed to the thermodynamic properties—of multi-electron batteries. Actually, the main disadvantages of current multi-electron systems are poor rate performance, large volume expansion and low reaction activity, which can be ascribed to the rich-cation structure and complex reaction pathway. In view of these inherent defects, various means are applied to improve kinetic performance (Fig. 5), such as preparing composite materials, structural design, morphology control, electrolyte design, storage mechanism optimization and external disturbance. These key materials and advanced technologies not only play a positive role in the reaction kinetics of multi-electron materials but also solve the problems of electrolyte stability, interface compatibility, anode safety and ion transport.

First, the composite materials can provide both high electrical and ionic conductivities to multi-electron materials (Fig. 5a) [87]. Graphene, carbon nanotubes, carbon dots, etc. with high electrical conductivities and different dimensions are used as composites to enhance the reaction activity and relieve volume change simultaneously [88]. Carbon materials with large sizes and high dimensional structures can form rapid transfer pathways for ions and electrons between isolated particles. Recently, the novel MXenes have been regarded as kind of superior composites because of the excellent electrical conductivity and rich superficial functional groups [89]. The negatively charged surfaces can facilitate the coupling of MXenes with other positively charged materials and prevent the aggregating with other composites. Moreover, the advanced conductive polymers can provide appreciable conductivities and effective interactions with multi-electron electrodes. It can exhibit strong adsorption capability for sulfur molecules to enhance cycling stability [90]. The ionic conductors as novel composite materials not only boost the transfer rate of cations but also prevent the continuous degradation of electrode and electrolyte [91]. The irreversible reactions of anionic redox cathodes or dual-ion batteries at high voltages can be suppressed by scavenging HF and H_2_O in organic electrolytes. In addition to multi-electron cathodes, the composite materials can be used as host materials to induce homogeneous electrodeposition of metal anodes [92]. For instance, the graphene oxides, MXenes, carbon nanosheets, etc. are composited with metal anodes to construct a metal affinity framework. On the other hand, the composite structure should be discussed to expound the influences of ordered and disordered modes. Hierarchical structures can usually achieve multiple functions, including ionic/electrical conductivity, interfacial compatibility, safety and structural stability [93], while the advanced composite architectures from 0D to 3D can combine the advantages of micro and macro structures synergistically [94].

Second, the crystal structure design is an effective avenue to reduce the diffusion barrier and enhance structural stability (Fig. 5b). Graphite is regarded as feasible host material for Li^+^ storage with high stability and low cost. However, the Na^+^, K^+^, and anions with a large radius are difficult to insert into graphite, thus the expanded layer spacing is designed to achieve rapid transfer of single large-sized ions or multiple small-sized ions [95]. This strategy has been applied to other 2D materials to attain a low diffusion barrier [96]. The kinetic advantages of two multi-electron materials can be combined in heterostructures, leading to a synergistic effect [57]. During the charge-discharge process, the irreversible intermediates of these two materials usually play an important role in accelerating ionic/electrical transport and enhancing structural stability. In particular, the heterostructures can form a built-in electric field to facilitate the charge migration [97]. Metal ion doping is an effective route to regulate atomic arrangement and electronic structure [98]. For example, when active metal ions are introduced into host materials, the dual-redox center can provide high capacity and fast reaction capability. Meanwhile, the optimized electronic structure reserves broad channels for ionic transfer and promotes metallic properties of materials. As mentioned above, the cation-rich structure is beneficial for cooperative transfer and high theoretical capacity due to the rational manipulation of defects and vacancies [85]. In addition to the diffusion kinetic, the interfacial transfer kinetic is also very important to multi-electron systems. The predominant crystal planes hold low adsorption energies for cations, thus induced growth methods have been applied to fully expose them [99]. These crystal planes also supply the open channels for insertion/extraction of cations.

Third, the morphology control of multi-electron electrodes can realize different sizes and various architectures, which is related to the contact areas and reaction sites between active materials and electrolytes (Fig. 5c). No matter what shape the materials, the morphology parameters of size, porosity, surface area and uniformity should be investigated to enhance the reaction dynamics and stability [100]. Overall, reasonable morphology parameters can balance the reaction activities of cation storage and electrolyte decomposition. Nanoscale or porous materials reserve buffer space for relieving volume expansion without significant decrease in volume energy density [101]. In practical application, the uniformity of materials decides the average electrochemical performances of high-energy density electrodes. With the development of advanced synthesis technology, various morphologies have been designed to achieve different electrochemical properties, including array structure, cage structure, nanocrystalline, cluster structure, etc. [102–104]. These micro-nano structures can be built in pure or composite materials. Some of them are prepared into self-supporting electrodes to further improve energy density and reaction stability [105]. Subsequently, the representative array, and cage structures with excellent mechanical properties and homogeneous nucleation sites are widely applied to the host materials for metallic anodes, which improve the deposition dynamics [106].

Fourth, the chemical composition and solvated structure of electrolytes are important factors for cations transfer (Fig. 5d). To increase the migration rate of cations in electrolytes, the viscosity, ion number and solvated structure are regarded as key factors. As shown in Fig. 1, aqueous electrolytes with low viscosity and high ionic conductivity can be used in intercalation-reaction, dual-ion and Li-O_2_ batteries to attain capability of rapid cation storage, and they are operated at moderate voltage ranges [107]. Meanwhile, with the development of solid electrolytes, the dynamic issues from electrolytes and interfaces attract a lot of attention. The composite solid electrolytes can balance the ionic migration and interfacial compatibility by combining organic and inorganic solid electrolytes [108]. Although the mobility of anions in electrolytes actively contributes to the ionic conductivity, the ion depletion layers may be formed on the anodic side due to the accumulation of anions, resulting in a poor dynamics [109]. Hence, single-ion electrolytes can limit the migration of anions to achieve high cation transference numbers close to 1 and stable interface on the anode. The solvated structure not only influences the migration rate of cations in electrolytes but also has a great effect on reaction dynamics of multi-electron electrodes. The co-intercalating of cations and solvents can facilitate fast ion transfer in host materials, especially for cations with a large radius [110]. Recently, the high concentration liquid electrolytes have been regarded as an effective means to enhance reaction dynamics in high-energy density batteries due to the special solvated structures, optimized interfacial properties and high chemical stability [111]. However, the low ionic conductivity, poor wettability and narrow electrochemical window should be solved in subsequent studies.

Fifth, more and more novel mechanisms have been proposed to enhance the reaction dynamics based on the multi-electron storage process (Fig. 5e). As mentioned above, the pseudocapacitance reaction can provide fast cation storage dynamics in intercalation, conversion and alloying reactions [112]. Similarly, the synergetic transfer effect of multi-ion in special materials with anisotropic channels also plays a positive role in cation diffusion [85]. It is worth mentioning that the rich defects on the surface or bulk of electrodes not only increase the electrical conductivity but also enhance the diffusion and mobility of cations [113]. These defects can be built via heteroatoms doping, plasma-induction etching, etc., which are applied to oxides, sulfides, polyanionic compounds, carbonaceous materials, etc. [114,115]. The weak interaction refers to the van der Waals force in 2D heterostructures, which is conducive to the rapid charge transfer and adjustable ion diffusion channels [116]. The special sandwich-like structure has been designed to obtain weak interaction layers with outstanding kinetic properties [117]. The cations with large radius (Na^+^ and K^+^) may cause huge volume expansion during the storage process in conversion and alloying electrodes, while the partial conversion reaction can be observed in these electrodes at fully discharged state, resulting in controllable deformation and stable structure [118]. The enhanced kinetic performance of high-energy density batteries is not only determined by the multi-electron cathodes but also influenced by the metal anodes. For metallic anodes, the metal-affinity framework can provide nucleation sites to reduce overpotential and induce homogeneous deposition [119]. These affinity frameworks are usually composed of noble metals, oxides or carbonaceous materials, which exhibit low energy to form lithiated compounds.

Sixth, a lot of other disturbances from both inside and outside need to be considered in order to analyze their influences on kinetic properties in practical applications (Fig. 5f). From the interior, the interfacial compatibility determines the ion transfer capability between electrolytes and electrodes. Hence, the artificial interphases are constructed to enhance ion transfer and improve cycling stability via *insitu* and *exsitu* methods [120]. The additives, fast ionic conductors, polymers and so on are used as feasible materials for artificial interphases [121]. For Li-S and Li-O_2_ batteries, the catalyst assistants can be utilized to enable reaction dynamics and improve reaction reversibility [4]. The metal oxides and sulfides are very effective catalysts to catalyze the redox kinetic of polysulfides, achieving fast and full conversion between soluble polysulfides and Li_2_S_2_/Li_2_S [122]. The solid catalysts and redox mediators are feasible approaches to improve the sluggish kinetics of oxygen reduction reaction and oxygen evolution reaction, especially for soluble catalysts [123]. From the exterior, the temperature regulation and magnetic field control have an important influence on the reaction kinetics of high-energy density batteries. For instance, dendrite-free Li growth behavior is observed at high temperature, which can be ascribed to the enhanced lithiophilicity and the increased Li ion diffusion coefficient [124], while the magnetohydrodynamics effect promotes ion transfer and uniform distribution of cations, which can be interpreted as Lorentz forces causing piral motion of cations [125]. In brief, these advanced approaches for attaining outstanding reaction kinetics in multi-electron processes and high-energy density batteries should be developed simultaneously, since the diversity and interaction exists in these complex systems.

## SOME FUTURE DIRECTIONS

**Figure 6.**
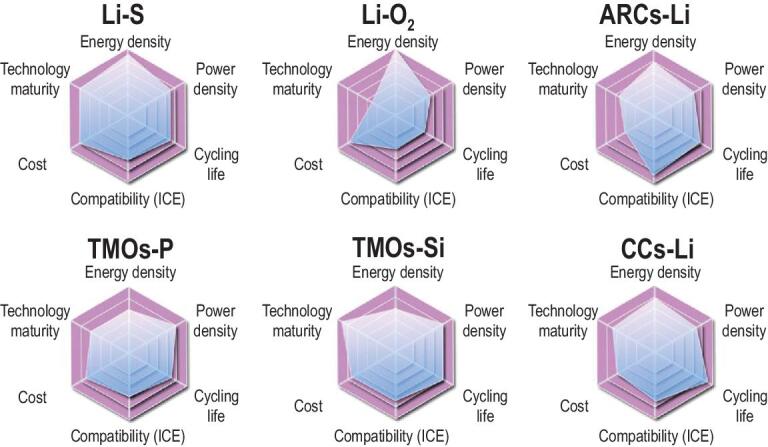
Evaluation of representative high-energy density batteries based on the multi-electron cathodes and metal anodes. ICE: initial coulombic efficiency.

Predictably, multi-electron electrodes gradually become the feasible route for developing high-energy density batteries to meet the actual demand of power supply. According to seven representative multi-electron reactions, more and more high-energy and long-life electrodes are prepared based on a series of advanced technologies. In particular, the anionic redox reactions and conversion reactions are regarded as feasible cation storage mechanisms for cathodes. Metals, silicon and phosphorus are considered superior anodes. As shown in Fig. 6, the Li-S and Li-O_2_ batteries exhibit outstanding advantages of energy density and low cost, which can be developed as next-generation energy storage technologies [4]. However, the poor conductivity and dissolution issue of electrodes should be solved via composite materials, catalyst assistants and solid electrolytes [126]. Furthermore, the electrodeposition dynamics of metallic anodes is the main limiting factor in high efficiency and safety. The layered metal oxides (TMOs) can be used as high-energy density cathodes with high technology maturity and long cycling life. When these cathodes are assembled with alloying P and Si anodes, the full cells can deliver considerable energy densities and high operating voltages [127]. Structural design and electrolyte optimization have enhanced the reaction kinetics of P and Si anodes to attain high rate performances. Although the TMOs with anionic redox reaction (ACRs) can increase the capacity and voltage, the reaction kinetics and redox reversibility of intermediate products limit their power densities. Moreover, the manufacturing cost of TMOs and ACRs will become crucial for large-scale application. In contrast, the cation-free cathodes based on the conversion reaction (CCs) provide excellent electrochemical properties and low cost, even at high temperature and rates [128]. Similar to the above systems, the cathode electrolyte interphases on CCs electrodes suffer from the poor stability and high ion transfer barrier. Fortunately, the pseudocapacitance effect and cooperative transfer effect can be developed to enhance reaction kinetics. In comparison, the technology maturity of sulfur, silicon, TMOs, CCs electrodes and ion-batteries are higher than that of phosphorus, ARCs electrodes and metal-batteries because of available raw materials and simple preparation methods. Currently, the practical prospects of TMOs-Si, TMOs-Li and Li-S batteries are better than other systems, indicating the promising application future for multi-electron electrodes and high-energy batteries. Furthermore, the multi-electron reactions based on the dual-ion systems and multivalent-ion systems (Mg^2+^, Ca^2+^, Al^3+^, etc.) are also regarded as viable candidates for building high-energy density batteries [129,130]. In future, the multi-electron reaction should be developed synergistically based on the new thermodynamic mechanism and optimized dynamic process.

## METHODS

The DFT calculations in this work were performed using the Vienna Ab-initio Simulation Package (VASP) [131]. The Blöchl's all-electron-like projector augmented plane wave (PAW) method was used to describe the interactions between ion cores and valence electrons [132]. The electron exchange-correlation interaction was treated using the generalized gradient approximation (GGA) with the Perdew-Burke-Ernzerhof (PBE) functional. Plane waves with a cutoff energy of 500 eV were used, and the 4 × 4 × 1 Monkhorst–Pack grid k-points were employed to sample the Brillouin zone integration. The structures were optimized until the energy and the force were converged to 1.0 × 10^−5 ^eV/atom and 0.02 eV/Å, respectively.
